# Effect of health systems strengthening in influencing maternal and neonatal health outcomes in Bungoma County, Kenya

**DOI:** 10.11604/pamj.2022.41.125.30170

**Published:** 2022-02-14

**Authors:** Beverly Marion Ochieng, Giorgia Lattanzi, Milka Choge, Dan Clement Owino Kaseje, Amardeep Singh Thind

**Affiliations:** 1 Tropical Institute of Community Health (TICH), Kisumu, Kenya,; 2 Hera-Right to Health and Development, Barcelona, Spain,; 3 United Kingdom Foreign, Commonwealth and Development Office, Nairobi, Kenya,; 4 Schulich School of Medicine and Dentistry, Western University, London, Canada

**Keywords:** Maternal, neonatal, care, health systems, access, utilization, quality

## Abstract

**Introduction:**

maternal and neonatal health status indicators have steadily improved over time in Kenya. Significant challenges remain, including persistent inequities between population subgroups, and a health system that delivers variable quality care and inconsistent access to care. This paper highlights results of an ex-post evaluation to assess the impact of maternal and health systems strengthening intervention to improve newborn health outcomes in Bungoma County, Kenya, focusing on access to and quality of maternal and neonatal care.

**Methods:**

the study design was quasi-experimental, using household surveys to assess outcomes at baseline and end-line. Stratified cluster sampling was used to identify households, based on heath facility catchment areas. Inclusion criteria were women aged 18-49. Chi-square and fisher´s exact tests were used. Patched-up design was used to compare outcomes before and after the intervention and intervention and control sub-counties.

**Results:**

provision of transport vouchers decreased barriers to accessto health care, resulting in an increased number of deliveries in health facilities. Women in the end-line group were 95% more likely to deliver at a health facility compared to 74% at baseline. The intervention improved potential and effective access to antenatal care as well as deliveries in health facilities. This positively impacted quality of care provision in the sub-counties.

**Conclusion:**

key elements of health system strengthening included reducing cost barriers and enhancing the capacity of the health facilities to deliver high quality care. The intervention addressed commonly identified supply-and demand-side barriers, providing stronger evidence that addressing these hindrances would improve utilization of maternal and child health services.

## Introduction

Kenya is estimated to have a population of 47.6 million [1]. Its health indicators (such as infant mortality rate, under-5 mortality rate, maternal mortality ratio, etc.) have steadily improved over the years, as evidenced by the 2008 and 2014 demographic surveys [2, 3]. This has been accompanied by its Universal Health Coverage (UHC) service coverage index being scored at 55, which is relatively high in the context of sub-Saharan Africa [4]. Despite this progress, many significant challenges remain, including persistent inequities between population subgroups, and a health care system that delivers variable quality of care and inconsistent access to care, especially for women and new-borns.

The country has responded to these challenges with a series of vigorous reforms in the past decade. The new 2010 constitution introduced 47 semiautonomous county governments, with substantial devolution of responsibility for health service delivery from the central government of these counties [5]. User fees for skilled delivery at health facilities were eliminated with the introduction of the Linda Mama Program. Reproductive Maternal Newborn Child Adolescent Health (RMNCAH) Investment Framework was launched in 2016 to guide smart, scaled up and sustained financing for delivering quality RMNCAH services [6]. The increasing focus of the Kenyan government on UHC (as one of the four pillars of its big four agenda) has brought much needed attention to maternal and child health indicators. However, persistent capacity gaps, especially at county health system level, stymie further improvement in RMNCAH goals. Newly created county governments often do not have the required technical capacity to manage devolved functions effectively, and there is lack of clarity around roles, responsibilities and structures existing at national and county level and flow of revenues and resources [7].

The United Kingdom Foreign, Commonwealth and Development Office (FCDO) funded the programme to reduce maternal and neonatal deaths in Kenya. In Bungoma, maternal neonatal health (MNH) program support commenced in 2013 and had two broad components, supporting health systems strengthening and demand creation, with a focus on maternal and new-born health services. Maternal and Newborn Improvement, Health Systems Strengthening (MANI-HSS) programme was implemented in 6 of the ten sub-counties. A range of activities were implemented at the county and sub-county level, in partnership with county health authorities. Activities included supporting health facilities by providing Emergency Obstetric and Neonatal Care (EmONC) training to staff and establishment of six technical working groups (TWGs) to address human resources for health, health care financing, quality of service delivery, monitoring and evaluation, community health services, and health products and technology management respectively.

At sub-county, activities included support for annual work plans and budgets, application of organisational capacity assessment tools, orientation and training of hospital management boards, support for health facility planning and budgeting, orientation and training of health facility management committees, and provision of travel vouchers for pregnant women to deliver at healthcare facilities [8] (Table 1). This paper highlights the results of the ex-post evaluation assessing the impact of the MANI HSS intervention in Bungoma County, with a focus on access and quality of maternal and neonatal care services.

**Table 1:** maternal and new-born improvement (MANI) programme intervention package

Intervention package	Intervention programme activities
Health systems strengthening	Leadership and governance: conducted policy dialogue meetings which resulted in the development of the county health policy, county health bill, county procurement policy, the new staff transfers policy, county annual work plans and budgets, annual review reports and application of the organizational capacity assessment (OCA) tool and capacity development plans; fora used were the monthly management breakfast meetings with county leadership, the set-up of technical working groups and a partner coordination forum; major focus included coaching and mentorship on leadership and governance of the CHMT; service delivery: focused on MNH - supportive supervision; performance review, mentorship; quality improvement; infection prevention and control; and referral systems
Demand creation for maternal and neonatal care services	Health financing: transport voucher system that was given to the women in intervention supported MANI sub counties; community support: including support to implementing the national community health strategy and more specifically establishing and strengthening community units (CU); support to Community Health Volunteers (CHV) and birth companions; social behavior change and communication; respectful maternity care; community scorecard and demand generation
Health workforce	Health service provider training and medical products, vaccines and technologies: included in-service and pre-service training in emergency obstetric and neonatal care (EmONC), quality improvement (QI) and maternal and perinatal death surveillance and response (MPDSR) training for hospital health service providers; leadership and governance -management training to hospital management boards; health facility management committees and support to planning and budgeting. Health service management training: technical working groups (TWGs) were created and operational, including for human resources for health, health care financing, quality of service delivery, monitoring and evaluation, community health services, and health products and technology management. Health information systems (HIS): at sub-county level support focused on the intervention sub-county annual work plans and budgets and application of the OCA with intervention sub-county teams as well as orientation and training of Hospital Management Boards (HMBs), micro support for health facility planning and budgeting and orientation and training of Health Facility Management Committees (HFMC)

CHMT: county health management team; MNH: maternal neonatal health; MANI: maternal and new-born improvement

## Methods

**Study setting:** Bungoma County is located in Western Kenya and has a population of approximately 1.5 million. The county comprises of ten sub-counties. Its per capita gross domestic product (GDP) is half that of the national average [9]. Bungoma lagged behind the national trend in key maternal health indicators. For example, with a Skilled Birth Attendant (SBA) rate of 40.8%, the demographic health survey 2014/15, ranked Bungoma 43^rd^ out of 47 counties in Kenya [3]. Women in Bungoma face significant social, economic and cultural challenges when accessing maternal and new-born health care. Most women stay at home to give birth, mostly as a result of lack of transport/money, lack of education, poor conditions at the health facility, and culture (i.e. importance of burying the placenta). High maternal and neonatal deaths were observed in the community, and poor health of newborns [8]. Bungoma presents a useful case for examining the effect of health systems strengthening on maternal and newborn health care given the poor maternal and child health indicators.

**Study population:** study population consisted of health facilities in Bungoma County, mothers from the catchment populations of the health facilities included in the survey who had delivered a baby during 12 months prior to the survey. Surveys were carried out by a team of researchers that was independent from the team involved in the intervention.

**Figure 1:**
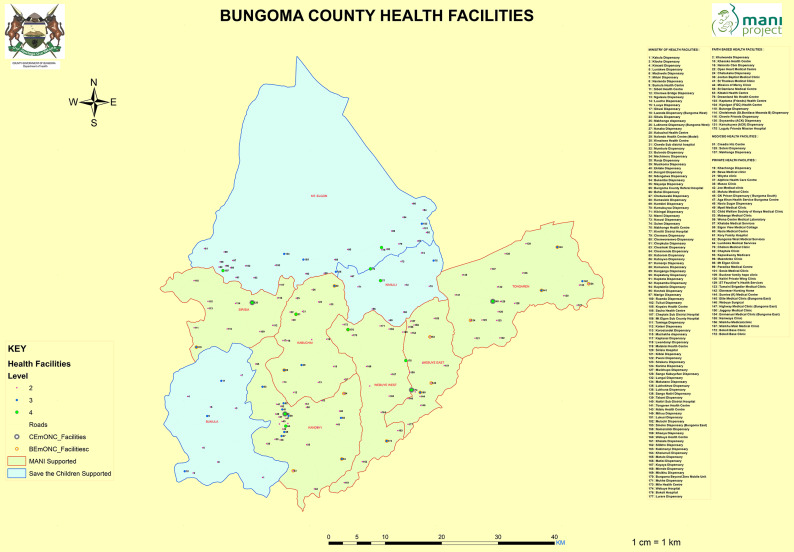
map of Bungoma County, highlighting health facilities and sub-counties supported by the MANI project and Save the Children International

**Study design:** an ex-post evaluation was conducted when the program had ended. The design had to be adapted to the available data at hand. The classic pre-post control group design requires baseline and end-line data for both intervention and control groups; the implementation of MANI program did not allow for this to be used [10]. MANI programme was implemented in six sub-counties (Kabuchai, Kanduyi, Sirisia, Tongaren, Webuye East and Webuye West); the remaining four sub-counties (Bumula, Mt. Elgon, Kimilili and Cheptais) were controls (Figure 1). At this stage, the design could be a static group comparison design (also known as the non-equivalent control group post-test only design) [11]. As selection is a major threat to the validity of this design, we attempted to control for it by using matching, as described below. In order to generate a sampling for the end-line surveys, eight health facilities in intervention sub-counties were matched with eight health facilities in the control sub-counties based on ownership, level, type and service provision characteristics. The service areas of each health facility were mapped using census data, and stratified cluster sampling was employed to identify households for interview. Respondent inclusion criteria were having delivered a live infant, a stillbirth or a third trimester spontaneous abortion within 12 months of the survey. A total of 448 women aged 18 - 49 years were interviewed in each of the two groups.

Availability of a pre-existing survey in the intervention areas allowed us to add a non-equivalent historical control for the intervention counties, which approximated a pre-test post-test design. Sample size for this baseline survey (conducted in 2015) was 478. Baseline survey questionnaire examined variables that assessed access utilization and quality of MNH services in intervention areas of the study. Questionnaires for both baseline and end-line surveys had been developed by adapting the demographic and health survey questionnaires to the indicators relevant to the MNH program and thus were comparable. In addition to demographics, questions focused on antenatal care attendance, delivery by skilled providers, access to care and perception of service quality and level of satisfaction with MNH services. Questionnaires were translated into Swahili and back translated into English for comparison with the original English version, to ensure accuracy of translation. The Swahili version was pretested in a population similar to the study population in a neighbouring sub county not involved in the survey.

Final design is depicted in Table 2 and described as a “patched up” design (10-17): O1 is the historical control (i.e. the pre-test, or the 2015 baseline) in the intervention counties, O2 is the post-test in the intervention counties, and O3 is the non-equivalent control. The ‘X´ depicts the intervention, and non-random (NR) signifies non-randomization [12]. It is important to note that the three groups (O1, O2 and O3) represent different samples. As recommended by Campbell, analysis of this design is based on comparing outcomes in the three groups. If O2 > O3 and O2 > O1 and both differences are statistically significant, we can state that there is an association of the intervention with the outcome [13]. As per Habicht *et al*. such congruency (of expected trends) classifies this design as 'plausible' (in their scale of adequate, plausible and probabilistic designs) [14].

**Table 2:** "patched up" study design

	2015		2018
NR	O1		
NR		X	O_2_
NR			O_3_

NR: non-random assignment; X: intervention; row 3 and 4 represent the intervention counties; row 5 represents the control counties

**Variables:** variables collected during surveys were grouped into five domains: a) mother´s potential access; b) mother´s actual access; c) baby´s actual access; d) quality of care received by the mother; and e) quality of care received by the baby.

**Potential access (mother):** costs are a recognized barrier to health care access, and interventions at the national level (elimination of user fees under the Linda Mama Scheme) and local levels (provision of transport vouchers as part of the intervention) aimed to decrease, if not eliminate, this barrier. The voucher scheme covered return transportation to the health facilities for the mother and one companion, which was provided by boda-boda (motorcycle taxi) drivers affiliated to the programme, thus reducing financial barriers to care. We assessed expenditures for antenatal care (ANC) services, transport to health facility, and delivery services as binary variables (zero, some expenditure).

**Actual access (mother):** the goal of interventions was to increase utilization of health services or improve the effective (i.e. realized) access. Maternal access to services was captured by asking women whether they had attended any ANC clinic during their last pregnancy, whether they had had at least 4 ANC contacts during the last pregnancy, whether an ANC contact had occurred in the first trimester, whether she had delivered in a health facility and whether she had received post-natal care (PNC) services within 48 hours of the delivery. We dichotomized all variables (no/yes).

**Actual access (baby):** was evaluated with a binary variable (no/yes) whether PNC was provided within 48 hours of birth.

**Quality of care (mother):** mothers were asked if they had delivered via C-section, and whether they were satisfied with the ANC care received. These variables were binary (no/yes). Quality of ANC was assessed by asking women about having received 14 clinical interventions or provider advice as part of service; ranging from measuring blood pressure to advice on nutrition. Thirteen or more positive answers were coded as high quality, 7 to 12 were coded as medium and six of less was coded as poor quality. Quality of PNC to the mother was assessed by whether they had received nine clinical interventions or provider advice as part of first PNC examination. These ranged from measuring blood pressure to advice about breastfeeding. Depending on the number of positive responses, this was coded as high (7 or more), medium (5-6) or low (4 or less).

**Quality of care (baby):** was captured with two variables. A dichotomous variable (no/yes) captured whether breastfeeding was initiated in the first hour. Quality of PNC provided to the child was assessed by asking mothers whether the child had received six clinical interventions or provider advice as part of the first PNC examination, ranging from physical examination to information about signs and symptoms that suggested that the baby was unwell. Six positive answers were coded as high quality, four and five as medium quality and three or less as low quality. All variables were categorical, we used Pearson´s chi-square or in cases of small numbers, fisher´s exact tests. Study participants provided written assent to participate. Study protocols were reviewed and approved by Nairobi University and Kenyatta National Hospital Ethics Review Boards. Ethical approval certificate Ref KNH -ERC/A/27.

## Results

**Respondents´ characteristics:** baseline (intervention only): a total of 478 women (age 18-49 years) were interviewed in 2015 baseline survey. Mean age was 25.9 years, and a majority had completed primary education (41%) or had no education/not completed primary education (36%). Mean wealth index score (on a 0 - 2.1 scale) was 0.85 (SD 0.1).

End-line (intervention and control): a total of 896 women (age 18-49 years) were interviewed, 448 from MANI and 448 from control areas. Mean age of respondents was 26.4 years in the intervention and 26.2 years in control area. More than a third (35%) of respondents had completed primary education in the intervention area; the corresponding number was 42% in the control area. Mean wealth index score (on a scale of 0-2.1) was 0.833 (SD: 0.230) in intervention and 0.901 (SD: 0.244) in control area.

**Before/after analyses (O1 vs O2):** Table 3 shows results of the baseline vs end-line analysis for the intervention group. Among the potential access variables, statistically significant change was noted for transport expenditure, with a greater proportion of women (39;16%) at end-line reporting zero expenditure compared to 0 (0%) at baseline. Statistically significant changes were noted for ANC services, with a greater proportion of women at end-line reporting at least once ANC contact, at least 4 ANC contacts (290; 74%), and first ANC contact in the first trimester (118; 30%), respectively, compared to the baseline figures of ANC four times (257; 54%), and first trimester ANC visit (100; 21%) respectively. Women in the end-line group were also more likely to deliver at a health facility (373; 95%) compared to 352 (74%) at baseline. However, children in the end-line group were less likely to get postnatal care within 48 hours as compared to the baseline group (Table 3).

**Table 3:** baseline vs end-line analyses

Indicator	Baseline		End-line	
	N	%	N	%
**Potential access (mother)**				
**Expenditure for ANC services**				
Zero	181	78	287	74
Some expenditure	52	22	102	26
Expenditure for transport to the health facility for delivery**				
Zero	0	0	39	16
Some expenditure	261	100	198	84
Expenditure for delivery**				
Zero	97	59	314	84
Some expenditure	67	40	58	16
**Actual access (mother)**				
At least one ANC contact with a formal provider**				
No	32	7	3	1
Yes	446	93	389	99
At least four ANC contacts with a formal provider**				
No	221	46	102	26
Yes	257	54	290	74
ANC contact in the first trimester of pregnancy**				
No	376	79	274	70
Yes	100	21	118	30
Delivery in a health facility**				
No	124	26	19	5
Yes	352	74	373	95
**Postnatal care provided to the mother within 48 hours of delivery**				
No	75	65	224	67
Yes	40	35	120	33
**Actual access (baby)**				
Postnatal care provided to the child within 48 hours of delivery**				
No	187	56	245	68
Yes	146	44	117	32
**Quality of care (mother)**				
High quality of antenatal care**				
No	124	28	46	12
Yes	321	72	343	88
Satisfaction with the antenatal care received**				
No	250	72	136	36
Yes	97	28	237	64
Type of delivery				
Caesarean section	31	9	26	7
Vaginal delivery	321	91	347	93
Quality of postnatal care provided to the mother**				
Low	86	57	48	38
Medium	31	21	66	53
High	33	22	11	9
**Quality of care (baby)**				
**Quality of postnatal care provided to the child**				
Low	9	36	31	26
Medium	8	32	37	31
High	8	32	51	43
Initiation of breastfeeding within the first hour**				
No	153	48	113	33
Yes	168	52	228	67

** p<0.05

Statistically significant changes were noted for quality variables. Women in the end-line group were more likely to report receipt of quality care, and more like to report satisfaction with ANC received, compared to baseline. A greater proportion of the end-line respondents reported initiation of breastfeeding in the first hour, and improved quality of postnatal care, as compared to the baseline group. No statistically significant differences were noted for personal expenditures for ANC services, receipt of postnatal care to mother within 48 hours, C-section rate and quality of post-natal care provided to the child between the two groups (Table 3).

**Intervention vs control analyses (O2 vs O3):** Table 4 shows results of the end-line survey analysis for the intervention and control group. Among the potential access variables, statistically significant change was noted for personal expenditure for ANC service and expenditure for transport to the health facility for delivery. A greater proportion of women in intervention areas reported no expenditure for ANC services and transport to the health facility to deliver respectively, compared to control areas (Table 4).

**Table 4:** intervention vs control group results

Indicator	Intervention		Control	
	n	%	N	%
**Potential access (mother)**				
Expenditure for ANC services**				
Zero	322	73	270	61
Some expenditure	121	27	172	39
Expenditure for transport to the health facility for delivery**				
Zero	42	15	8	3
Some expenditure	237	85	229	97
**Expenditure for delivery**				
Zero	361	86	320	82
Some expenditure	61	14	70	18
**Actual access (mother)**				
**At least one ANC contact with a formal provider**				
No	4	1	7	2
Yes	444	99	441	98
**At least four ANC contacts with a formal provider**				
No	114	25	134	30
Yes	334	75	314	70
**ANC contact in the first trimester of pregnancy**				
No	316	71	329	73
Yes	132	29	119	27
Delivery in a health facility**				
No	25	6	58	13
Yes	423	94	390	87
Postnatal care provided to the mother within 48 hours of delivery**				
No	258	61	172	44
Yes	165	39	218	56
**Actual access (baby)**				
Postnatal care provided to the child within 48 hours of delivery**				
No	254	88	130	73
Yes	36	12	47	27
**Quality of care (mother)**				
Quality of antenatal care**				
No	205	46	243	55
Yes	239	54	198	45
**Satisfaction with the antenatal care received**				
No	47	11	33	7
Yes	397	89	408	93
**Type of delivery**				
Caesarean section	29	7	29	7
Vaginal delivery	394	93	363	93
Quality of postnatal care provided to the mother**				
Low	70	42	120	55
Medium	83	50	84	39
High	12	7	14	6
**Quality of care (baby)**				
Quality of postnatal care provided to the child**				
Low	45	27	27	11
Medium	56	34	118	47
High	63	38	105	42
**Initiation of breastfeeding within the first hour**				
No	119	31	101	29
Yes	266	69	253	71

** p<0.05

Statistically significant changes were noted for health facility delivery, with a greater proportion of women in intervention areas reported having delivered in a health facility than in control areas. However, women and children in the intervention areas were less likely to receive postnatal care within 48 hours as compared to the control sites; this finding was statistically significant. Statistically significant changes were also noted for quality of care variables. Women in intervention areas were more likely to report receipt of high quality ANC care (239; 54%) compared to 178 (45%) at control sites, and receipt of better quality maternal postnatal care, compared to women in to the control areas. Better quality child service provision was reported in control areas compared to intervention areas. A greater proportion of mothers in control areas reported a postnatal check-up of their infants than in project areas. A larger proportion of mothers in control areas reported high quality PNC child services compared to intervention areas. These differences were statistically significant.

No statistically significant differences were noted between MANI intervention and control areas for personal expenditure for delivery, ANC contact in first trimester of pregnancy, at least four ANC, satisfaction with the antenatal care received, C-section rate, initiation of breastfeeding within the first hour, at least three postnatal contacts for the child within the first two months and satisfaction with the maternity care received (Table 4).

Table 5 presents results in a summative manner. The '+' indicates that the relationship is significant and positive (in the expected direction); the ‘-‘ indicates a statistically significant relationship but in the opposite direction. A blank cell denotes that the relationship was not statistically significant. A variable that is statistically significant in both analyses is taken as an indicator of change possibly associated with the intervention. Based on this decision rule, the intervention possibly had a role in improving potential and actual access to antenatal care and health facility delivery services for mothers as well as positively impacting quality of care provision (Table 5).

**Table 5:** overall summative results

MNH Indicators	Baseline vs end-line (O1 vs O2)	Intervention vs control (O2 vs O3)
**Potential access (mother)**		
Expenditure for ANC services		+
Expenditure for transport to the health facility for delivery	+	+
Expenditure for delivery	+	
**Actual access (mother)**		
At least one ANC contact with a formal provider	+	
At least four ANC contacts with a formal provider	+	
First ANC contact in the first trimester of pregnancy	+	
Delivery in a health facility	+	+
Postnatal care provided to the mother within 48 hours of delivery		_
**Actual access (baby)**		
Postnatal care provided to the child within 48 hours of delivery	-	_
Quality of care (mother)		
Quality of antenatal care	+	+
Satisfaction with the antenatal care received	+	
**Caesarean section delivery**		
Quality of postnatal care provided to the mother	+	+
Quality of care (baby)		
Quality of postnatal care provided to the child		-
Initiation of breastfeeding within the first hour	+	

## Discussion

Study has shown that key elements of health system strengthening included reducing cost barriers and enhancing capacity of health facilities to deliver the high quality care in maternal and child health. Provision of transport vouchers decreased barriers to accessing care resulting to an increased number of deliveries in health facilities. The intervention improved access to antenatal care and health facility delivery for mothers positively impacting quality of care provision.

The intervention had an impact on transport expenditure, as focused provision of transport vouchers was a key element of the intervention. Beneficiaries of the voucher scheme were poorer women identified in intervention communities, who were reached by a community health worker with information on birth preparedness and received a transport voucher in their third trimester of pregnancy. Voucher scheme covered return transportation to the health facilities for the mother and one companion, which was provided by boda-boda (motorcycle taxi) drivers affiliated to the programme, thus reducing financial barriers to care. In addition, by making a reliable and fast means of transportation available for free to women, the intervention also addressed the second delay in the three delays model, i.e. delay in accessing care, which can further contribute to improving maternal and neonatal health [15]. Women were able to reach health facilities in a more timely manner thus reducing the second delay.

In terms of realized access, the intervention seemed to have facilitated deliveries in health facilities. Intervention activities included demand creating activities such as re-orienting traditional birth attendants into birth companions, health education sessions aimed at changing views on skilled birth attendance, and community scorecard dialogues aimed at promoting social accountability, our finding is eminently plausible. However, it is wise to keep in mind changes in the contextual environment that could have also played a role. A major environmental factor was the abolition of user fees for maternity care, enacted by the government in 2016. Literature suggests that this led to the increase of facility based deliveries from 44% (in 2012/13) to 62% (in 2016) [16]. Our study design did not allow us to disentangle these factors. Taken together, the voucher and the user fee elimination are key to enhancing social justice, especially when placing a priority on the most vulnerable, and makes eminent economic sense [17].

The intervention package also seemed to have had a positive impact on quality of care, as measured by quality of ANC and PNC received by mothers; however, our results indicate that it did not have the same impact on effective access for the baby, where we found a negative impact on whether postnatal care was provided to the baby within 48 hours of delivery. This could be plausible for three reasons. First, it is possible that the MANI intervention with its goal of health systems strengthening (e.g. providing EmONC training and refreshers, TWG focusing on service delivery, application of organizational capacity assessment tools, etc.) could have had a greater focus and emphasis (especially in implementation) on mothers compared to their babies, thus resulting in a differential impact.

Second, literature indicates that while women are generally able to report accurately on aspects of postnatal care received, indicators related to new-born care received by the child are subject to greater recall bias than those related to postnatal care received by the mother [18]. A recent review of population-based survey data on maternal and newborn care in 20 sub-Saharan Africa countries showed that approximately two-thirds (65%) of women and their babies had received some form of postnatal care, only 3% reported receiving all seven interventions included in the analysis [19].

Third, it is important to keep in mind the context of Bungoma, where several projects of the MNH Programme´s County Innovation Challenge Fund (CICF) were implemented at the same time as the MANI HSS project. Most notably, two projects focusing on neonatal care by Save the Children International (SCI) and by Mount Kenya University (MKU) were active in both intervention and control sub-counties. In addition, the Liverpool School of Tropical Medicine (LSTM) was implementing its training programme ‘Making it Happen´ (MiH) in the region [8]. In this dynamic environment, marginal end-user changes that could be attributed to the MANI HSS project would be difficult to detect, especially with our design.

While our study design was quasi-experimental, with a focus on improving utilization and access to maternal and child care health services, measurements were compared to a systematic review covering sub-Saharan Africa [20], and several qualitative studies in Kenya [21,22] whose purposes were mainly to identify factors influencing access and utilization of skilled delivery at health facilities. Study among pastoralist women in Kenya [21] identified distance, poor roads and difficulty of obtaining and paying for transport as hindering factors on the demand side; quality of treatment and disrespectful and unfriendly care offered at health facilities on the supply side, comparing to our study which tested the effectiveness of an intervention package that addressed the commonly identified barriers, providing stronger evidence that, addressing these hindrances, would enhance utilization of maternal and child health services. Our study addressed factors that hindered access and utilization to maternal and health care services and created demand among the pregnant women and mothers. The limitations in our study design suggests the need to test the intervention package by a more complete quasi-experimental or a randomized controlled design with the newborn services equally targeted, to improve attribution of the outcomes to the intervention package.

A study conducted in Kenya [22] identified poverty, lack of transport, and poor treatment by nurses as some of the barriers in the utilization of maternal and child health services. Unlike this study, our study intervened and offered services that enabled mothers and pregnant women access services which, in turn, improved health indicators in intervention areas. Cited studies [20-22] identified poverty, lack of transport, and poor treatment by health care workers as major barriers in accessing maternal and child care. These studies identified the same barriers addressed in our article, but based on observational research designs, while ours demonstrated improvement in utilization and access of care based on a quasi-experimental design hence stronger evidence on improving maternal and child health outcomes. Our study tested the effectiveness of an intervention addressing identified barriers on demand and supply sides and providing stronger evidence that, addressing these hindrances, would enhance utilization of maternal and child health services, hence improve health outcomes.

A major limitation of our study is the weak design, which does not completely eliminate threats to validity of selection, interaction of selection with maturation, and selection with history. To re-iterate, this is at best a weak plausible design, which can only show that the intervention is associated with the observed differences (and are not causal). Given the limitations of the study design there is need to test the intervention package by a more complete quasi-experimental study design.

We are also limited in terms of data and its availability. Baseline data for the intervention sub-counties was limited to an existing survey and to ensure comparability we were restricted to the same questions (and responses) used in the existing survey. In addition, the policy environment and geographic context in which the interventions were implemented was not static, making clear attribution challenging due to a multiplicity of programmes.

## Conclusion

The intervention had an impact on improving access to care and delivering better quality of care to pregnant mothers in Bungoma. Key elements include reducing cost barriers to generate demand and enhancing the capacity of the health facilities to deliver moderately improved quality of care.

### What is known about this topic

- *Access to delivery services is hindered on demand side by lack of income, lack of transport and lack of birth preparedness on the demand side and quality of care in terms of waiting time and provider client interpersonal relationships;*
- *Poverty, lack of transport, and poor treatment by nurses as some of the barriers in the utilization of maternal and child health services;*
- *Distance, poor roads and difficulty in obtaining transport as hindering factors and quality of treatment and disrespectful and unfriendly care offered at health facilities*.

### What this study adds

- *Reducing cost barriers by transport voucher, and providing means of transport is effective in generating demand;*
- *Abolition of maternity fees is effective in generating demand for health facility skilled deliveries;*
- *Enhancing capacity of health facilities to deliver moderately improved quality of care had a positive impact on utilization of skilled delivery*.
